# Can we know more about atherosclerosis in cyanotic patients with congenital heart disease—the potential role of sphingosine-1-phosphate?

**DOI:** 10.3389/fcvm.2025.1531136

**Published:** 2025-04-28

**Authors:** Sonia Alicja Nartowicz, Marcin Gabriel, Magdalena Janus, Artur Cieślewicz, Katarzyna Malesza, Agnieszka Bartczak-Rutkowska, Maciej Lesiak, Olga Trojnarska

**Affiliations:** ^1^ 1st Department of Cardiology, Poznan University of Medical Sciences, Poznań, Poland; ^2^ Department of General and Vascular Surgery, Poznan University of Medical Sciences, Poznań, Poland; ^3^ Department of Clinical Pharmacology, Poznan University of Medical Sciences, Poznań, Poland

**Keywords:** endothelial function, cyanosis, congenital heart disease, atherosclerosis, S1P

## Abstract

**Background and aims:**

Progress in cardiology has extended the lifespan of patients with congenital heart defects (CHD). Cyanotic patients are exposed to typical diseases of adulthood, including atherosclerosis. Rheological changes typical of cyanosis affect the vascular endothelium's function and may promote atherosclerosis development. We assessed the endothelial function and its relationship to biochemical parameters, particularly sphingosine-1-phosphate, in cyanotic CHD patients.

**Method:**

A cross-sectional study including 36 adult CHD cyanotic patients [(12 males) (39 median, 19–73 years)] with arterial blood oxygen saturation less than 92% and 30 healthy controls [(11 males) (38.5 median, 26–59 years)] was performed. All patients underwent clinical examination, blood sampling, and ultrasonography, during which endothelial function was assessed using intima-media thickness (IMT) and flow-mediated dilatation (FMD).

**Results:**

We did not demonstrate any difference between CHD patients and the control group in the IMT complex and FMD. Patients with cyanosis are characterized by higher S1P serum levels (*p* = 0.04), lower ApoM (*p* = 0.04), and HDL concentrations (*p* = 0.02). Only FMD correlated positively with HDL cholesterol (*p* = 0.02) concentration. The IMT complex correlates positively only with BMI (*p* = 0.04). No factor was statistically significant in the multiple logistic regression model for FMD <6.5%.

**Conclusions:**

The values of the analyzed biochemical and clinical factors (except for the reduced HDL fraction), the lack of inflammatory factor activity, and the increased S1P concentration indicate the dominance of antiatherosclerotic activity in this population. FMD and IMT are preserved, which suggests that the risk of early atherosclerotic changes in this group is comparable to the remaining population.

## Introduction

**Figure 1:**
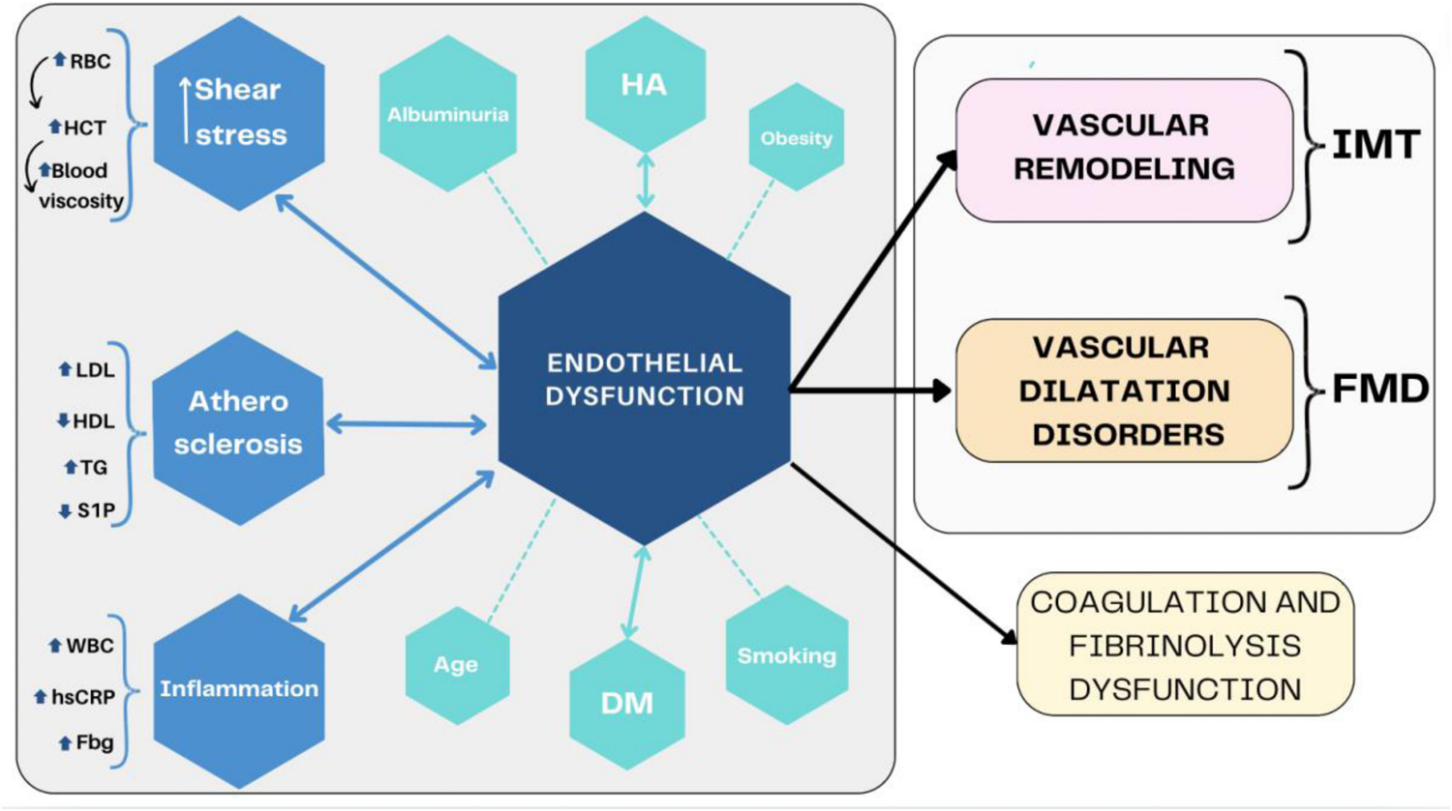
Endothelial dysfunction summary. DM, diabetes mellitus; Fbg, fibrinogen; FMD, flow-mediated dilatation; HA, hypertension; HCT, hematocrit; HDL, high-density lipoprotein; hs-CRP, high-sensitivity C-reactive protein; IMT, intima-media thickness; LDL, low-density lipoprotein; S1P, sfingozyno-1-phosphorus; TG, triglycerides; WBC, white blood cells.

The development of modern cardiology and cardiac surgery has resulted in cyanotic patients with congenital heart defects (CHD) reaching adulthood. Among them are patients with complex primary cyanotic defects, whose repair operations usually leave some complications of the procedure, causing desaturation or remnants of the defect. There are also those with leak defects that were not operated on in childhood. Consequently, all of them are exposed to diseases associated with the aging process, including atherosclerosis (1). Due to the significantly changed rheological conditions as a consequence of their typical erythrocytosis (Figure 1) and at the same time a small number of this population, data on this condition are scarce and contradictory. The first reports published on this subject by Perloff et al., who, based on anatomopathological analysis and angiography of coronary arteries, showed their significant dilatation and the absence of atherosclerotic plaques, suggested the lack of atherosclerotic complications in this group of patients (2). Some publications appear later to support this opinion (3–6), while other authors do not confirm it (7, 8). At the same time, studies on this state conducted on the general population provide more and more information on the pathogenesis of atherosclerosis and, consequently, the possibility of non-invasive assessment of the risk and the degree of its advancement. Many of them, such as non-invasive assessment of the structure of the vessels, biochemical analysis, and parameters indicating the inflammatory process, have already been used in studies on cyanotic patients (4–6, 9–12). To our knowledge, sphingosine-1-phosphoran (S1) and apolipoprotein M (apoM) concentrations were analyzed only in one multi-center study (10). By surveying a comparably large group of patients observed in one academic center, we set ourselves the following goal: to assess the structure and function of arterial vessels and their relationship to selected clinical and biochemical phenomena, with particular emphasis on sphingosine-1-phosphate in cyanotic patients with congenital heart defects in search of early signs of atherosclerosis.

## Material and methods

### Studied group and control group

We included thirty-six patients (24 females) with cyanosis secondary to CHD (Table 1) with a median age of 38 years (range 19–73) with less than 92% arterial blood oxygenation (Table 2). All patients under the care of our Adult Congenital Heart Disease Outpatient Clinic did not meet the exclusion criteria and agreed to participate in the study. The matched control group consisted of 30 healthy volunteers (19 females) with a median age of 38.5 (range 26–59). Every patient was examined during one visit in the morning before the pharmacotherapy dose between January and September 2023. The baseline characteristics of the group are summarized in Table 2. We excluded patients with acute and chronic inflammatory disease (in the preceding 3 months), coexistence of Down syndrome, and concurrent malignancies. Approval of the local bioethical committee was acquired. According to the ethical guidelines in the Declaration of Helsinki of 1964, informed consent was obtained from all participants.

**Table 1:** Characteristics of congenital heart defects.

Characteristics of cyanotic patients with congenital heart diseases	
Eisenmenger syndrome	16 (44.6%)
ASD, *n* (%)	6 (16.7%)
ASD and VSD, *N* (%)	1 (2.8%)
VSD, *n* (%)	4 (11.1%)
PDA, *n* (%)	2 (5.6%)
PDA and VSD, *n* (%)	1 (2.8%)
EA and ASD, *n* (%)	1 (2.8%)
cc-TGA and VSD, *n* (%)	1 (2.8%)
PA, VSD and MAPCA's, *n* (%)	5 (13.9%)
ToF, *n* (%)	4 (11.1%)
Univentricular heart, *n* (%)	11 (30.5%)

ASD, atrial septal defect; cc-TGA, congenitally corrected transposition of the great arteries; EA, Ebstein anomaly; MAPCA's, major aortopulmonary collateral arteries; PDA, patents ductus arteriosus; VSD, ventricular septal defect.

**Table 2:** Baseline characteristics of the study and control group.

Baseline characteristics/additional diagnosis/ medications/Framingham score	Cyanotic patients (*n* = 36)	Control group (*n* = 30)	Cyanotic patients vs. control group (*p*-value)
Baseline characteristics			
Male: female	12:24	11:19	0.8
Age (years)	38 (19–73)	38.5 (26–59)	0.9
Oxygen blood saturation (%)	87.5 (65–91)	99 (96–99)	**<0** **.** **001**
Body mass index (kg/m^2^)	22 (15.4–43)	24 (18.8–36)	**0** **.** **02**
Systolic blood pressure	107.5 (90–160)	122.5 (100–175)	**<0** **.** **001**
Diastolic blood pressure	75 (60–100)	80 (60–100)	0.3
Additional diagnosis			
Smokers, *n* (%)	2 (5.6)	3 (10)	0.5
Diabetes mellitus, *n* (%)	2 (5.6)	0 (0)	0.2
Stroke, *n* (%)	4 (11.1)	0 (0)	0.06
Overweight, *n* (%)	7 (19.4%)	9 (30)	0.3
Obesity, *n* (%)	1 (2.8)	1 (3.3)	0.9
Medications			
Statins, *n* (%)	4 (11.1)	2 (6.7)	0.5
Advanced therapy for pulmonary arterial hypertension, *n* (%)	14 (38.9)	0 (0)	**0** **.** **001**
Endothelin receptor antagonist, *n* (%)	13 (36.1)	0 (0)	**0** **.** **001**
Phosphodiesterase 5 inhibitor, *n* (%)	12 (33.3)	0 (0)	**0** **.** **001**
Prostcyclin analogs, *n* (%)	1 (2.8)	0 (0)	**0** **.** **001**
Framingham 10-year CVD risk score^a^			
Low <10%, *n* (%)	25 (69.4%)	18 (60%)	*p* = 0.7^a^
Medium 10–19%, *n* (%)	1 (2.8%)	3 (10%)	
High ≥20%, *n* (%)	2 (5.6%)	1 (3.3%)	
Patients <30 years-old, *n* (%)	8 (22.2%)	8 (26.7%)	

CVD, cardiovascular heart disease; diam, diameter; IVS, intraventricular septum; LA, left atrium; LV -left ventricle; LVEF, left ventricle ejection fraction; LW, lateral wall; PW, posterior wall; RV, right ventricle; TRV, peak tricuspid regurgitant velocity. Bold values are statistically significant.

^a^ Framingham 10-year CVD risk score is suitable only for patients older than 30; younger patients were omitted.

### Clinical data and risk factors

The baseline data were collected, including resting heart rate, systolic and diastolic blood pressure, weight, and height. During the study, data were also collected regarding the history of smoking, hypertension, hyperlipidemia, diabetes, and history of myocardial infarction or stroke in the patient and his first-line family members. The Framingham Risk Score (F-score) was calculated based on the collected data on cardiovascular disease (CVD) risk factors (13). The derived 10-year risk of developing CVD divided patients into three subgroups: low (<10%), medium (10%–19%), and high-risk (>20%) groups (13).

### Laboratory method and blood sampling

Blood sample collection was performed in the morning at least 14 h after the last meal. Local standard laboratory methods and assays measured the lipoproteins, hematology, and inflammatory markers. Blood samples for plasma S1P and apoM assessment were centrifuged and frozen at −80°C after material collection until assayed. ApoM and S1P levels were measured using enzyme-linked immunosorbent assay (ELISA) kits by the manufacturer's recommendations. The ApoM intra-assay coefficient of variation was <8%, and the inter-assay coefficient of variation was <10%. The ApoM test disclosed values as low as 39 ng/ml. The S1P intra-assay coefficient of variation was <4.8%, and the inter-assay coefficient of variation was <7.9%. The ApoM test disclosed values as low as 39 pg/ml.

### Vascular and endothelial function

Every patient had a carotid artery (CA) assessment (14). Intima media thickness (IMT) of the carotid artery was measured with the 7–12 MHz transducer (Logic 7, GE ultrasound machine, Fairfield, CT, USA. The images visualizing the near and far walls of the common carotid artery during diastole to ensure that the transducer was transecting the artery at 90° were used to demonstrate the best IMT. One observer interpreted all scans using a quantitative analysis package (Siemens, Berlin, Germany), giving an axial resolution of 0.001 mm. The measurements of IMT were made over 10 mm segments and at 1 mm intervals of the near and far walls of the left and right common carotid arteries to determine the maximal and mean values of IMT. Flow-mediated dilatation (FMD) was assessed in every individual during the morning examination in resting supine positions in a temperature-controlled room (20–22°C), as previously reported (15). The ultrasounds and standard brachial artery measurements were made using a 7–12 Mhz linear array transducer, Logic 7, GE. The same occlusive cuff protocol assessed the flow-mediated dilatation. The vessel diameter response to reactive hyperemia (FMD) was calculated from the diameter brachial artery dimension (BAD) measured immediately before cuff inflation and expressed as a percentage change.

### Statistical analysis

Calculations were performed using the Statistica 13 program from TIBCO. Data were presented as the mean value with standard deviation (±SD) and range of minimal-maximal value with a median. The level of significance was *α* = 0.05. The result was considered statistically significant when *p* < α. The normality of the distribution of variables was tested using the Shapiro–Wilk test. To compare variables between the two groups, the Student's *t*-test for unrelated samples (when normal distribution and equal variances), Welch's test (when variances were not equal), or Mann–Whitney test (when there was no normal distribution) were performed. Univariable regression analyses were applied for each potential cofounder to identify possible confounding factors. In the final multivariable regression model, including FMD <6.5% (16), as an outcome, factors with a *p*-value <0.1 were included. The participant's (control/patients) type was included as an independent variable of interest, while age, sex, and BMI (potential confounders) were covariates. We did not perform such a model for IMT because there were too few incorrect results for this measurement.

A different team member was responsible for each part of the study: clinical examination with echo, carotid and brachial ultrasound, blood sampling with laboratory analysis, and statistical analysis, which eliminated any possible bias in the patient assessment.

## Results

The demographic and clinical characteristics of the patients are summarized in Table 2. The Framingham Risk Scor (F-score) comparison presented therein did not show any differences between the patients and the control group. Vascular function is summarized in Table 3. We did not demonstrate any difference between the study and control groups in measurements performed using the brachial artery (BAD: *p* = 0.1), and flow-mediated dilatation percentage (*p* = 0.6); assessment of the carotid artery showed no difference in the measurements of IMT (*p* = 0.9).

**Table 3:** Vascular function characteristics.

Parameter	Cyanotic patients (*n* = 36)	Control group (*n* = 30)	Cyanotic patients vs. control group (*p*-value)
BAD	0.3 (0.02–0.5)	0.4 (0.3–0.5)	0.1
FMD %	8.8 (2–29.2)	9.3 (2.4–34.6)	0.6
IMT	0.06 (0.04–0.09)	0.06 (0.04–0.1)	0.9

BAD, brachial artery dimension after ischemia; FMD, flow mediated dilatation; IMT, intima-media thickness.

### Biochemical markers

The biochemical characteristics of the study and control groups are summarized in Table 4. There were no differences in inflammatory markers between CCHD patients and the control group (white blood cells: *p* = 0.7; hs-CRP: *p* = 0.07; fibrinogen: *p* = 0.7). The study group was characterized by a reduced platelet count [151.5 ×10^9^/L (57–339) vs. 212 ×10^9^/L (171–319); *p* < 0.001]. The CCHD patients had lower total cholesterol (4.1 mmol/L ±0.9 vs. 4.75 mmol/L ±0.5; *p* = 0.003) and high-density lipoprotein (HDL) levels (1.3 ±0.3 mmol/L vs. 1.6 mmol/L ±0.4; *p* = 0.02), while the low-density lipoprotein (LDL; *p* = 0.08) and triglycerides (*p* = 0.5) were no different in comparison to controls. There was significantly higher level of total bilirubin [21.5 (7.4–83.3) vs. 14.5 (4.1–25.3); *p* < 0.001], unconjugated bilirubin [14.7 (4.1–60.1) vs. 10.8 (2.1–18.3); *p* < 0.001], and conjugated bilirubin [7 (2.3–58.1) vs. 4.4 (2.3–9); *p* < 0.001] in patients with cyanosis. This group also had higher S1P [213.9 pg/ml (107.3–2,832.4) vs. 179 pg/ml (95.5–300.8); *p* = 0.04] and lower apoM [278.5 ng/ml (260.4–308) vs. 301.9 ng/ml (290.3–308.6); *p* = 0.02]. The creatinine (*p* = 0.9) and albumin levels (*p* = 0.7) levels did not different significantly between analyzed groups.

**Table 4:** Biochemical characteristics of the study and control group.

Parameter	Cyanotic patients (*n* = 36)	Control group (*n* = 30)	Cyanotic patients vs. control group (*p*-value)
Baseline characteristics			
Hematocrit (%)	0.5 (0.35–0.73)	0.41 (0.33–0.47)	**<0** **.** **001**
Hemoglobin (mmol/L)	11.1 (±2.1)	8.7 (±0.8)	**<0** **.** **001**
Red blood cells (10^12^/L)	5.85 (±1.3)	4.5 (±1.1)	**<0** **.** **001**
Platelets (10^9^/L)	151.5 (57–339)	212 (171–319)	**<0** **.** **001**
White blood cells (10^9^/L)	5.5 (2.8–9.9)	6.1 (4–9.9)	0.7
Total cholesterol (mmol/L)	4.1(±0.9)	4.75 (±0.5)	**0** **.** **003**
LDL cholesterol (mmol/L)	2.6 (±0.9)	3.0 (±0.8)	0.08
HDL cholesterol (mmol/L)	1.3 (±0.3)	1.6 (±0.4)	**0** **.** **02**
Triglicerydes (mmol/L)	1.1 (0.5–2.3)	1.1 (0.6–2.2)	0.5
Creatinine (umol/L)	75.5 (48–396)	77.5 (57–107)	0.9
hsCRP (mg/L)	2 (2–20)	2 (2–26)	0.07
Fibrinogen (mg/dl)	275.2 (±69.2)	270.2 (±46.5)	0.7
ApoM (ng/ml)	278.5 (260.4–308)	301.9 (290.3–308.6)	**0** **.** **04**
S1P (pg/ml)	213.9 (107.3–2,832.4)	179 (95.5–300.8)	**0** **.** **04**
Albumin (g/L)	40.5 (27–48)	41 (36–49)	0.7
Total bilirubin (umol/L)	21.5 (7.4–83.3)	14.5 (4.1–25.3)	***p*** **<** **0.001**
Unconjugated bilirubin (umol/L)	14.7 (4.1–60.1)	10.8 (2.1–18.3)	***p*** **<** **0.001**
Unconjugated bilirubin (umol/L)	7 (2.3–58.1)	4.4 (2.3–9)	***p*** **<** **0.001**

APTT, activate partial thromboplastin time; ApoM, apolipoprotein M; INR, international normalized ratio; HDL, high density lipoprotein; hsCRP, hidgh sensitivity C-reactive protein; LDL, low density lipoprotein; NTproBNP, N-terminal prohormone of brain natriuretic peptide; PT, prothrombin time; S1P, sfingozyno-1-phosphorus.

Bold values are statistically significant.

### Correlation of vascular parameters with analyzed clinical data

As shown in Table 5 among all the markers of hypoxia, inflammation, and biochemical indicators of atherosclerosis, IMT is influenced only by BMI (*r* = 0.4; *p* = 0.04). With FMD%, the increase in HDL cholesterol concentration affected the FMD% value rise (*r* = 0.4; *p* = 0.02).

**Table 5:** Univariate correlations of vascular parameters with clinical and imaging data in the patients with cyanosis secondary to congenital heart disease.

Parameter	FMD%	IMT
Age	*r* = 0.2	*r* = 0.1
	*p* = 0.4	*p* = 0.5
SpO2	*r* = 0.1	*r* = 0.2
	*p* = 0.5	*p* = 0.3
F-score	*r* = 0.3	*r* = 0.03
	*p* = 0.08	*p* = 0.9
BMI	*r* = −0.07	*r* **=** **0.4**
	*p* = 0.7	***p*** **=** **0.04**
RBC	*r* = −0.2	***r* = −0.04**
	*p* = 0.3	*p* = 0.8
Hbg	*r* = −0.2	*r* = 0.02
	*p* = 0.3	*p* = 0.9
HCT	*r* = −0.2	*r* = −0.03
	*p* = 0.3	*p* = 0.9
TC	*r* = 0.3	*r* = 0.2
	*p* = 0.1	*p* = 0.3
LDL	*r* = 0.04	*r* = 0.2
	*p* = 0.8	*p* = 0.4
HDL	***r* =** **0.4**	*r* = 0.2
	***p*** **=** **0.02**	*p* = 0.2
TG	*r* = 0.04	*r* = 0.2
	*p* = 0.8	*p* = 0.3
S1P	*r* = −0.3	*r* = −0.07
	*p* = 0.07	*p* = 0.7
ApoM	*r* = 0.3	*r* = 0.05
	*p* = 0.08	*p* = 0.8
Fibrinogen	*r* = −0.2	*r* = 0.1
	*p* = 0.3	*p* = 0.6
WBC	*r* = 0.03	*r* = 0.3
	*p* = 0.3	*p* = 0.4
hsCRP	*r* = 0.2	*r* = 0.06
	*p* = 0.2	*p* = 0.4

ApoM, apolipoprotein; BAD, brachial artery dimension after ischemia; BMI, body mass index; FMD, flow mediated dilatation; F-score, Framingham risk score; Hbg, hemoglobin; HCT, hematocrit; HDL, high density lipoprotein; hsCRP, high-sensitivity C-reactive protein; IMT, intima-media thickness; LDL, low density lipoprotein; RBC, red blood cells; S1P, sfingozyno-1-phosphorus; SpO2, blood oxygen saturation, TC, total cholesterol; TG, triglycerides; WBC, white blood cells. Bold values are statistically significant.

### Multiple logistic regression

None of the analyzed parameters were statistically significant in the univariable and multilogistic regression model, with FMD <6.5% (Table 6).

**Table 6:** Multiple logistic regression analyses for factors potentially associated with FMD <6.5%.

Parameter	Unadjusted model		Multivariable model	
	*β* ± SE	*p*-value	Exp(B)	*p*-value
Type of participant (control	0.09 ± 0.5	0.9	2.1	0.3
Age	−0.04 ± 0.02	0.1	1.0	0.5
Sex (male)	−0.5 ± 0.5	0.4	0.5	0.2
BMI	0.01 ± 0.05	0.9	1.0	0.6
SpO2	0.0 ± 0.3	0.9		
RBC	0.04 ± 0.2	0.8		
Hbg	0.04 ± 0.1	0.7		
HCT	0.4 ± 2.6	0.9		
TC	−0.04 ± 0.3	0.9		
LDL	0.04 ± 0.3	0.9		
HDL	−0.5 ± 0.7	0.5		
TG	0.3 ± 0.6	0.7		
S1P	0.0 ± 0.1	0.1	1.0	0.1
Fibrinogen	0.01 ± 0.01	0.08	1.0	0.1
D-dimer	0.0 ± 0.1	0.3		
WBC	−0.05 ± 0.2	0.8		
hsCRP	0.1 ± 0.07	0.1	1.1	0.2

BMI, body mass index; Exp(B), exponential value of B; FMD, flow mediated dilatation; HDL, high density lipoprotein; Hbg, hemoglobin; HCT, hematocrit; hsCRP, high-sensitivity C-reactive protein; LDL, low density lipoprotein; RBC, red blood cells; SE, standard error; S1P, sfingozyno-1-phosphorus; SpO2, blood oxygen saturation, TC, total cholesterol; TG, triglycerides; WBC, white blood cells.

## Discussion

The adult cyanotic patients with CHD we studied were characterized by a similar Framingham Risk Score for coronary artery disease in comparison to healthy controls. Rheological changes typical for cyanosis, related to hypoxemia and progressive erythrocytosis, disturb the nitric oxide (NO) secretion, resulting in increased blood viscosity, causing dysfunction of the endothelium, which indirectly affects vascular function (7, 17). Our study showed that the most important parameter illustrating endothelial function in conduit arteries obtained noninvasively in patients with cyanosis in the course of CVD – endothelial-dependent FMD, was not different from that observed in the healthy population. Similar results, based on a multicenter study comparable in size to ours, were presented by Tarp et al. and in 45 patients by Petersen et al. (8, 10). Significantly lower values of this parameter in the group of interest to us were observed by other authors (3, 18, 19). In both univariate and multivariate analyses, we did not demonstrate that any clinical and biochemical parameters we examined influenced the increased risk of atherosclerosis, defined in large populations by a decrease of FMD by lower than 6.5% (16).

There were also no differences between the values of ultrasound imaging of carotid IMT between the study and the control group, which allows for assessing early atherosclerotic changes and indicates the risk of their development. Our predecessors achieved similar results and found no differences between cyanotic CHD patients and controls in coronary artery calcification score, carotid plaques, or maximal carotid plaque thickness (3, 20). The work of Duffels et al. performed on 44 patients showed significantly lower IMT values, which the authors pathophysiologically associate with low total cholesterol levels because it is, as has been demonstrated in the general population, related to low intima-medial thickness (5). Such heterogeneous results do not allow for drawing pathophysiological conclusions regarding the population of interest to us, especially since this parameter positively correlates only with BMI, a generally recognized atherogenic factor.

The physiology of the vessels studied in atherosclerosis is influenced by morphological elements of the blood and biochemical changes that we analyzed. The parameters of the red blood cell system constituting cyanosis's essence were significantly increased. The number of platelets, however, is lower, consistent with most studies, and is one of the pathophysiological justifications for the absence of atherosclerosis in this population (6, 21). As many researchers have demonstrated (3–6), this concept is confirmed by reduced cholesterol levels, explained by the genetic determinants of cyanosis, hypoxemia, erythrocytosis, and related factors. Our study showed significantly lower levels of total cholesterol and the HDL fraction, a factor limiting the formation of atherosclerotic plaques. However, we observed no differences in the LDL and triglyceride levels.

Nevertheless, the literature data are inconsistent: reduced total cholesterol levels are described by our predecessors, indicating a pathophysiological similarity to hypocholesterolemia caused by hypoxemia in people living at high altitudes, in whom atherosclerosis is rare (2, 6, 22). The authors mentioned above, like Fyfe et al., also report a low level of the LDL fraction (3–6). Reduced concentrations of triglycerides were observed by Martínez-Quintana et al. (23). However, the authors of the multi-center study by Tarp et al., comparing cyanotic patients to the control group, did not observe any differences in the total cholesterol concentration or any of its fractions, apart from a decrease in the HDL concentration in the studied group (20). Fyfe et al. also confirmed our observation of a reduced HDL concentration, explaining this phenomenon by the activity of several rare genetic disorders (6). Our study also showed a relationship between this cholesterol fraction and FMD, suggesting the importance of HDL in reducing the risk of atherosclerosis in this population. However, this concept was not confirmed by the multivariate analysis we conducted.

The concentration of bilirubin, including unconjugated bilirubin, assessed by us in the studied population was significantly higher than in the control group. The cause of this phenomenon in patients with cyanosis is the intensive degradation of heme in the erythrocytosis typical of this disease (2, 24). Unconjugated bilirubin is an endogenous antioxidant inhibiting LDL cholesterol oxidation and reducing atherosclerotic risk (25). Another phenomenon that could potentially “protect” cyanotic patients from atherosclerosis is a decrease in the number of platelets, which our study confirmed in comparison to the control group and is consistent with other reports (26, 27). It is known that low platelet counts are antiatherogenic, and platelet counts are typically low or thrombocytopenic in adults with cyanotic CHD. The cause of thrombocytopenia is megakaryocytes shunting from the systemic venous circulation into the systemic arterial circulation so that they cannot shed platelets by cytoplasmic fragmentation in the pulmonary vascular bed.

Our study did not demonstrate increased inflammatory activity; leukocytes, CRP, and fibrinogen levels did not differ from those observed in healthy people. Oechslin et al. also noted a lack of deviations in the level of leukocytes (18). Differently, Tarp et al. obtained increased CRP and leukocyte levels (20). Therefore, it is difficult to draw clear conclusions based on the presented results regarding the significance of the inflammatory process in atherogenesis in the studied population.

The substance currently focusing the attention of researchers dealing with atherosclerosis is sphingosine-1-phosphate (S1P) - a robust transmitter acting on five types of receptors located on the cell membrane. It has been observed that its concentration in patients with coronary artery disease is significantly reduced (28). It has been proven that by causing a pleiotropic effect through phosphorylation of NO, it causes vasodilation, reduces the inflammatory reaction, prevents apoptosis, and seals the endothelial cell membrane, limiting myocyte proliferation. However, this compound also has an unfavorable effect on vessels, intensifying prothrombotic processes. This dualism results from the relationship and cooperation of S1P with the transmitters and types of receptors necessary for its activity. A detailed analysis of these phenomena exceeds the scope of the publication. However, we know that most (60%) of S1P binds to HDL, which is transported by ApoM and has an anti-atherosclerotic effect. ApoM also modulates the activity of this sphingolipid (28). When combined with albumin, the remaining part of S1P intensifies atherosclerotic processes (28). The observation of S1P concentration in cyanotic patients seems particularly justified in the context of the still undefined pathophysiology of arterial vessels and contradictory data on atherosclerotic changes in this population, and the fact that the S1P, apart from platelets and endothelium, is produced in erythrocytes, which are particularly numerous in cyanosis.

Our study showed that the S1P concentration was significantly higher than the control group, suggesting that prothrombotic-atherogenic processes may dominate in cyanotic patients. However, this thesis was not confirmed in further analysis, as we did not observe any correlation between S1P and parameters describing the structure (IMT) and the function (FMD) of vessels. The concentration of apoM we assessed in the studied population was significantly lower than in the control group. Similar results of the S1P and apoM levels were obtained by Tarp et al. in the only study to date evaluating these substances in the cyanotic population (20). The lack of “parallelism” correlation between both substances in the general population was also described by other authors who speculate that S1P is also transferred in various proportions by albumins or that apoM transfers other lipids (retinol) (28). So many unknowns do not allow for drawing clear conclusions regarding the significance of sphingosine-1-phosphate in the pathogenesis of atherosclerotic changes in cyanotic patients with CHD. However, characteristic of chronic hypoxia erythrocytosis undoubtedly justifies further studies, which may shed a different light on the unresolved problem of atherosclerosis in this population.

## Limitations

The main limitation of our study is the small number of participants and the significant variation in cardiac anatomy. However, these are limitations typical for studies involving patients with congenital heart defects, and in particular with cyanosis. Additionally, cyanosis in some of the study participants is the result of primary cyanotic defects. In contrast, in others, it is secondary to pulmonary hypertension in the course of shunt defects, the pathophysiology of which is different.

## Conclusions

The values of most of the analyzed biochemical factors (except for the reduced HDL fraction), the lack of activity of inflammatory factors, and the increased concentration of sphingosine-1-phosphate indicate the dominance of antiatherosclerotic activity in cyanotic patients with congenital heart disease. Also, brachial artery FMD and intima-media thickness are preserved in this population, which suggests that the risk of early atherosclerotic changes in this population is comparable to that of the remaining population. However, further studies are necessary, focusing primarily on factors directly related to erythrocytosis resulting from hypoxia, an example of which is sphingosine-1-phosphate.

## Data Availability

The original contributions presented in the study are included in the article/Supplementary Material, further inquiries can be directed to the corresponding author.
